# Postsynaptic SDC2 induces transsynaptic signaling via FGF22 for bidirectional synaptic formation

**DOI:** 10.1038/srep33592

**Published:** 2016-09-15

**Authors:** Hsiao-Tang Hu, Hisashi Umemori, Yi-Ping Hsueh

**Affiliations:** 1 Institute of Molecular Biology, Academia Sinica, Taipei, 11529, Taiwan; 2 Department of Neurology, F.M. Kirby Neurobiology Center, Boston Children’s Hospital, Harvard Medical School, Boston, MA 02115, USA

## Abstract

Functional synapse formation requires tight coordination between pre- and post-synaptic termini. Previous studies have shown that postsynaptic expression of heparan sulfate proteoglycan syndecan-2 (SDC2) induces dendritic spinogenesis. Those SDC2-induced dendritic spines are frequently associated with presynaptic termini. However, how postsynaptic SDC2 accelerates maturation of corresponding presynaptic termini is unknown. Because fibroblast growth factor 22 (FGF22), a heparan sulfate binding growth factor, has been shown to act as a presynaptic organizer released from the postsynaptic site, it seems possible that postsynaptic SDC2 presents FGF22 to the presynaptic FGF receptor to promote presynaptic differentiation. Here, we show that postsynaptic SDC2 uses its ectodomain to interact with and facilitate dendritic filopodial targeting of FGF22, triggering presynaptic maturation. Since SDC2 also enhances filopodial targeting of NMDAR via interaction with the CASK-mLIN7-MINT1 adaptor complex, presynaptic maturation promoted by FGF22 further feeds back to activate NMDAR at corresponding postsynaptic sites through increased neurotransmitter release and, consequently, promotes the dendritic filopodia-spines (F-S) transition. Meanwhile, via regulation of the KIF17 motor, CaMKII (activated by the NMDAR pathway) may further facilitate FGF22 targeting to dendritic filopodia that receive presynaptic stimulation. Our study suggests a positive feedback that promotes the coordination of postsynaptic and presynaptic differentiation.

During neural development, synapse formation is one of the critical steps for the assembly of neuronal circuits. How pre- and post-synaptic termini coordinate and synchronize bidirectional differentiation is a critical issue. Transmembrane proteins that mediate transsynaptic interactions, such as neurexin–neuroligin^1^,^2^,^3^,^4^, N-cadherin^5^,^6^,^7^, Eph-Ephrin^8^,^9^,^10^ and the leucine-rich repeat transmembrane (LRRTM)^11^, have been shown to function bidirectionally for synapse formation and maturation. In this report, we found that secreted fibroblast growth factor 22 (FGF22) and postsynaptic syndecan-2 (SDC2) protein complex generate a positive feedback machinery to control bidirectional differentiation of synapses.

SDC2, a transmembrane heparan sulfate proteoglycan, is highly concentrated at dendritic spines^12^,^13^. The heparan sulfate part of SDC2 interacts with extracellular matrix proteins and growth factors^14^,^15^. Consequently, SDC2 is able to act as an adhesion molecule to regulate cell adhesion and as a coreceptor to facilitate signaling by presenting growth factors to the specific growth factor receptors^14^,^15^,^16^. In neurons, SDC2 expression levels are increased during development, which concurs with synapse formation *in vitro* and *in vivo*^13^,^17^. Knockdown of endogenous SDC2 reduces the density of dendritic spines in mature cultured neurons^18^. Interestingly, overexpression of SDC2 in immature neurons mimics intrinsic dendritic spinogenesis. SDC2 overexpression at 1–2 days *in vitro* (DIV) triggers robust dendritic filopodia formation, followed by a filopodia-spines (F-S) transition, and then by dendritic spine maturation at least one week earlier than for the intrinsic process^13^,^18^, strengthening the role of SDC2 in dendritic spinogenesis.

The molecular regulation of SDC2 in spinogenesis has been dissected. Interaction of the cytoplasmic conserved motif 1 (C1) of SDC2 and neurofibromin is required for dendritic filopodia formation, i.e. the initial stage of dendritic spinogenesis^18^,^19^. The C2 motif of SDC2 interacts with syntenin^20^, CASK^12^ and synbindin^21^. Via the interaction with CASK, SDC2 further associates with mLIN7 and NMDAR in the filopodia-forming stage, and promotes the targeting of these proteins to filopodial tips. The SDC2-CASK-mLIN7-NMDAR protein complex is critical for the morphological change from filopodia to spines, i.e. the F-S transition^22^. Moreover, CASK also links SDC2 to the protein 4.1-F-actin cytoskeleton to stabilize SDC2-induced dendritic spines^23^ (summarized in Fig. 1a).

Postsynaptic SDC2 also promotes presynaptic formation because SDC2-induced dendritic spines frequently associate with presynaptic synaptophysin^18^. However, the mechanism of transsynaptic signaling induced by SDC2 is unknown. Fibroblast growth factor 22 (FGF22) acts as a presynaptic organizer secreted from postsynaptic sites to promote presynaptic differentiation^24^,^25^,^26^. More specifically, FGF22 initiates organization of excitatory (glutamatergic) synapses in the hippocampus^25^. The motor proteins KIF17 and KIF3A are involved in excitatory synaptic targeting of FGF22^26^. KIF17 also controls synaptic targeting of NMDAR through the CASK-mLIN7-MINT1 tripartite complex^27^. Similar to other FGF family members, FGF22 possesses a conserved region for interaction with heparan sulfate, so it is very possible that FGF22 binds SDC2 and mediates the transsynaptic signalling of SDC2. Here, we used cultured hippocampal neurons to investigate this possibility.

## Results

### Postsynaptic SDC2 promotes pre- and post-synaptic differentiation

**Figure 1:**
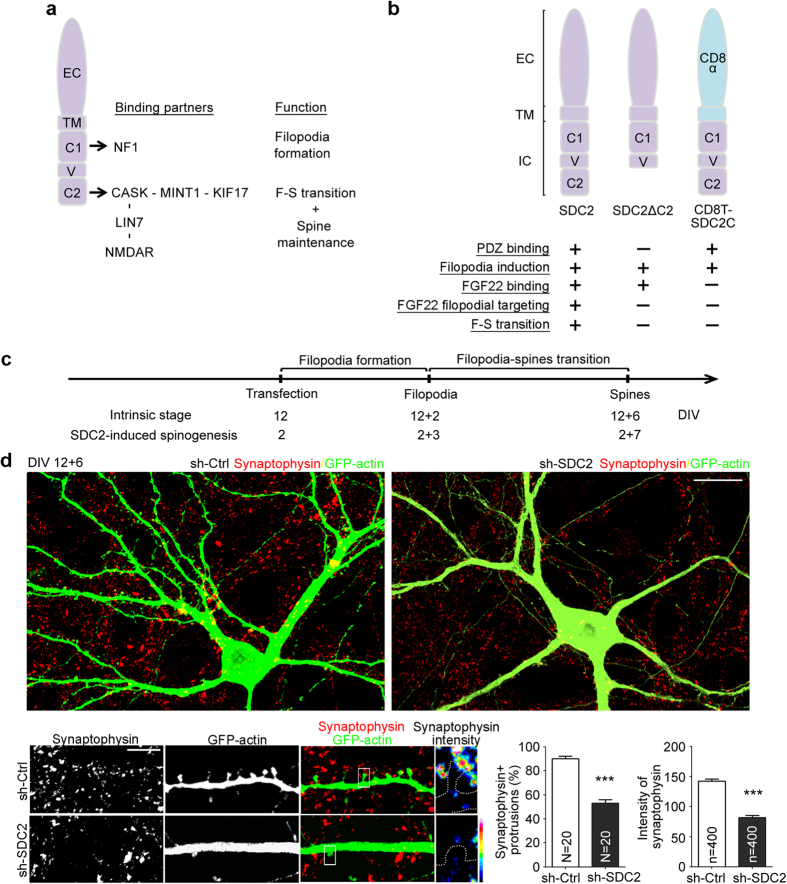
SDC2 is required for presynaptic maturation. (**a,b**) Schematic summary of SDC2-interacting proteins and functions. (**a**) Cytoplasmic domain of SDC2 and its known interacting proteins and their corresponding functions. The conserved domain 1 (C1) and 2 (C2) and the variable region (V) are indicated. C1 interacts with neurofibromin; C2 directly binds the PDZ domain of CASK. CASK then interacts with mLIN7-NMDAR and MINT1-KIF17. (**b**) Summary of the domain structure and functions of SDC2, SDC2ΔC2 and CD8T-SDC2C based on literature and the results of this report. EC: extracellular domain; TM: transmembrane domain; IC: intracellular domain. (**c**) Flow chart of the experimental design. Both the intrinsic developmental process and SDC2-induced spinogenesis of cultured rat hippocampal neurons are indicated. Neurons were co-transfected with various plasmids at 2 or 12 days *in vitro* (DIV) and subjected to immunostaining 3, 6 or 7 days later, as indicated, to monitor dendritic filopodia and spine formation. (**d**) Compared with non-silencing control sh-Ctrl, expression of the SDC2 knockdown construct sh-SDC2 decreases association of presynaptic synaptophysin with dendritic spines in mature neurons. The heat maps show the intensities of synaptophysin. Both whole cell and enlarged images are shown as indicated. N, number of analyzed neurons; n, number of analyzed protrusions. Samples were collected from two independent experiments. Data represent the mean plus SEM. ***P < 0.001. Scale bar: (**d**) whole cell image, 20 μm; enlarged inset, 5 μm.

For this report, SDC2 knockdown and several various expression constructs were used to study the role of SDC2 in presynaptic maturation (Fig. 1b). Under our culturing conditions, mature dendritic spines are typically formed after around 18 days *in vitro* (DIV). To monitor or manipulate intrinsic dendritic spine formation, transfection was usually performed at 12 DIV and immunostaining was carried out at 18 DIV (Fig. 1c, intrinsic stage). The role of SDC2 in presynaptic maturation of the intrinsic developmental stage was first evaluated by RNA knockdown in mature neurons. Similar to our previous findings^18^, knockdown of SDC2 using a previously-established knockdown construct (sh-SDC2^18^) reduced dendritic spine density compared with a non-silencing control sh-Ctrl (Fig. 1d). Note that remaining spines in SDC2 knockdown neurons showed a decrease in the percentage of synaptophysin-positive dendritic protrusions, as well as a lower intensity of synaptophysin surrounding the tips of dendritic protrusions at 18 DIV (Fig. 1d). These data suggest that postsynaptic SDC2 regulates both postsynaptic spine formation and presynaptic differentiation.

**Figure 2:**
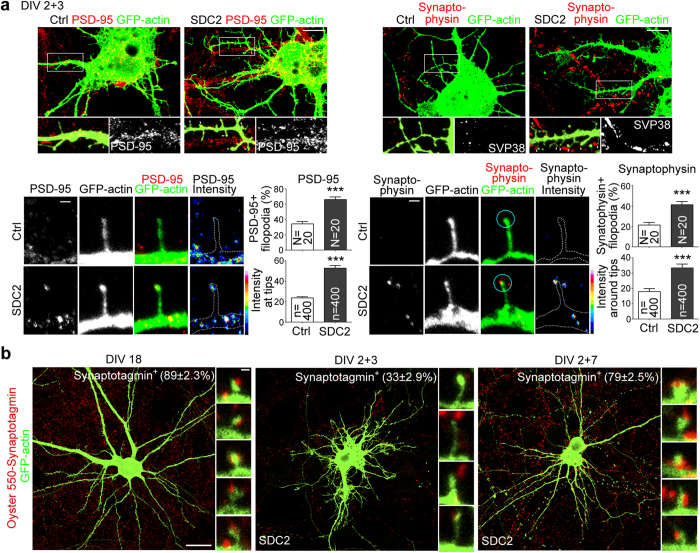
Postsynaptic expression of SDC2 promotes both pre- and post-synaptic maturation. (**a**) SDC2-induced accumulation of PSD-95 (left panel) and synaptophysin (SVP38, right panel) at or adjacent to filopodia compared with a vector control. Whole cells, enlarged dendrite fragments and individual dendritic filopodia are shown as indicated. In the images of enlarged dendritic fragments, white arrows indicate dendritic protrusions containing PSD-95 or adjacent synaptophysin. In the right panel, circles indicate the regions for synaptophysin quantification. Heat maps show the intensities of indicated proteins. N, number of analyzed neurons; n, number of analyzed protrusions. (**b**) Synaptotagmin antibody uptake assay. Cultured rat hippocampal neurons were transfected with GFP-actin alone at 12 DIV or a combination of GFP-actin and SDC2 at 2 DIV and subjected to synaptotagmin antibody uptake assay at 18 (left), 5 (middle) and 9 DIV (right). Mature neurons at 18 DIV were used as a positive control for synaptotagmin antibody uptake assays. The experiment of 5 and 9 DIV showed that postsynaptic SDC2 promotes presynaptic vesicle recycling at both dendritic filopodia- and spine-forming stages. The percentages of synaptotagmin antibody-positive protrusions are indicated. Samples were collected from two independent experiments. A total of 20 neurons and 400 protrusions for each group were analysed. Data represent the mean plus SEM. ***P < 0.001. Scale bar: (**a**) whole cell image, 10 μm; enlarged inset, 1 μm; (**b**) whole cell image, 20 μm; enlarged inset, 0.5 μm.

To further confirm that postsynaptic SDC2 is actively involved in presynaptic maturation, SDC2 transfection was performed at 2 DIV, which induces dendritic filopodia formation at 5 DIV and dendritic spine formation at 9 DIV (Fig. 1c, SDC2-induced spinogenesis). At 5 DIV, we noticed that the postsynaptic marker, PSD-95, tended to accumulate at tips of SDC2-induced dendritic filopodia (Fig. 2a, left panel). Because PSD-95 is an important adaptor of NMDAR, filopodial distribution of PSD-95 is consistent with our previous finding that NMDAR is enriched at the tips of SDC2-induced filopodia^22^. In addition to this postsynaptic marker, we found that SDC2 also effectively recruited the presynaptic marker synaptophysin adjacent to postsynaptic filopodia (Fig. 2a, right panel). In the filopodial tips containing PSD-95 or adjacent to synaptophysin puncta (defined by an intensity >20 units), the intensities of individual PSD-95 and synaptophysin puncta were also greater in SDC2-expressing neurons compared with vector controls (Fig. 2a). Together, these results suggest that even at the dendritic filopodia-forming stage, postsynaptic expression of SDC2 can accelerate the accumulation of both post- and pre-synaptic markers at synaptic contact sites.

To investigate whether postsynaptic SDC2-induced presynaptic termini are functional, we performed a synaptotagmin antibody uptake assay^28^,^29^, which is based on the rationale that active presynaptic termini contain internalized synaptotagmin labeled with specific antibody. We first used mature hippocampal neurons to validate the specificity of synaptotagmin antibody uptake at synaptic sites. Neurons were transfected with GFP-actin at 12 DIV to outline morphology and incubated with Oyster-550-conjugated synaptotagmin antibody for 1 hr at 18 DIV. As expected, the signals of synaptotagmin antibody were punctate and adjacent to the majority (~90%) of dendritic spines of mature hippocampal neurons (Fig. 2b, left panel). We then applied synaptotagmin antibody to SDC2-transfected neurons at 5 and 9 DIV. In general, we found fewer synaptotagmin puncta at 5 DIV compared to with those at 9 and 18 DIV (Fig. 2b, middle panel vs. left and right panels), consistent with the expectation that neurons at 5 DIV were still immature. For SDC2-transfected neurons at 5 DIV, similar to synaptophysin, synatotagmin puncta were adjacent to the tips of SDC2-induced filopodia (Fig. 2b, middle panel). We found that ~40% and ~33% of SDC2-induced filopodia were synaptophysin- and synaptotagmin antibody-positive, respectively, at 5 DIV (Fig. 2a, right panel vs. Fig. 2b, middle panel). It suggests that ~80% of presynaptic termini that contact with SDC2-induced filopodia are able to release neurotransmitters during a 1-hr antibody incubation period. At 9 DIV, ~80% of SDC2-induced protrusions were adjacent to synaptotagmin, which was similar to that of mature neurons at 18 DIV (Fig. 2b, left panel vs. right panel). Taken together, these results suggest that postsynaptically-expressed SDC2 is able to induce presynaptic differentiation, even in immature cultured neurons.

### SDC2 binds and facilitates FGF22 targeting to dendritic filopodia and spines

**Figure 3:**
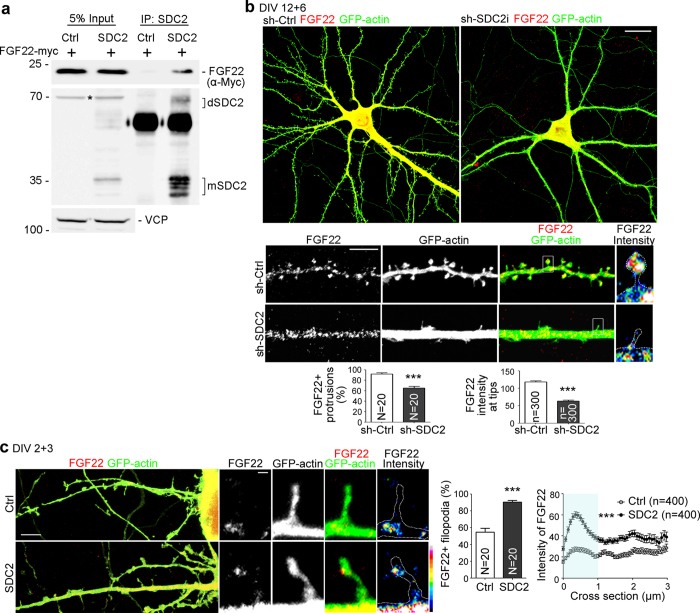
SDC2 facilitates FGF22 targeting to dendritic filopodia and spines. (**a**) Coimmunoprecipitation of SDC2 and FGF22-myc. Neuro2A cells were cotransfected with indicated constructs and subjected to immunoprecipitation and immunoblotting using the indicated antibodies. Because of heparan sulfate modification and SDS-resistant dimerization via the transmembrane domain, multiple SDC2 bands are present in SDS-PAGE. dSDC2: SDC2 dimer; mSDC2: SDC2 monomer. Asterisk indicates non-specific signal. (**b**) Knockdown of SDC2 in mature neurons reduces the synaptic distribution of FGF22-mCherry. Whole cells and enlarged dendrite fragments are shown as indicated. The heat maps of individual protrusion show the intensities of FGF22-mCherry. (**c**) FGF22-mCherry is distributed to the tips of SDC2-induced filopodia. The results of the percentages of FGF22-positive filopodia and line scanning from the tips of filopodia to dendritic shafts are shown. The fluorescence intensities at tips indicated by the light blue area in the chart were used for statistical analysis. Data represent the mean plus SEM. N, number of analyzed neurons; n, number of analyzed protrusions. Samples were collected from two independent experiments. ***P < 0.001. Scale bar: (**b**) whole cell, 20 μm; dendrite, 5 μm; (**c**) lower magnification, 10 μm; high magnification, 1 μm.

Because FGF22 acts as a presynaptic organizer released from postsynaptic sites^24^ and because, like other FGF members, FGF22 contains a conserved heparan sulphate binding motif, we wondered whether SDC2 binds and presents FGF22 to presynaptic sites and, thus, induces presynaptic maturation. First, the interaction between SDC2 and FGF22 was confirmed by immunoprecipitation. In Neuro2A cells, immunoprecipitation using SDC2 antibody that recognizes the ectodomain of SDC2^18^ readily precipitated SDC2 as well as FGF22 (Fig. 3a), indicating a physical association between SDC2 and FGF22.

We then investigated whether FGF22 is targeted to the filopodial tips via SDC2. In the intrinsic developmental process, around 90% of dendritic spines of sh-Ctrl-transfected neurons were FGF22-positive at 18 DIV (Fig. 3b). Knockdown of endogenous SDC2 at 12 DIV noticeably reduced the FGF22-positive dendritic spines to less than 70% at 18 DIV (Fig. 3b). Moreover, SDC2 knockdown also reduced the intensity of FGF22 to around 50% compared with a non-silencing control (Fig. 3b). In immature neurons, more than 90% of SDC2-induced dendritic filopodia contained FGF22 aggregates (Fig. 3c). Furthermore, FGF22 was concentrated at the tips of these filopodia (Fig. 3c). There were much fewer dendritic filopodia in neurons that were transfected with a vector control^22^ and, of these spontaneously-formed filopodia, even fewer were FGF22-positive and the intensity of FGF22 at the filopodial tips was also noticeably lower (Fig. 3c). Taken together, these results suggest that SDC2 facilitates dendritic filopodial and spine targeting of FGF22.

### FGF22 mediates the transsynaptic signal of SDC2 to promote presynaptic differentiation

**Figure 4:**
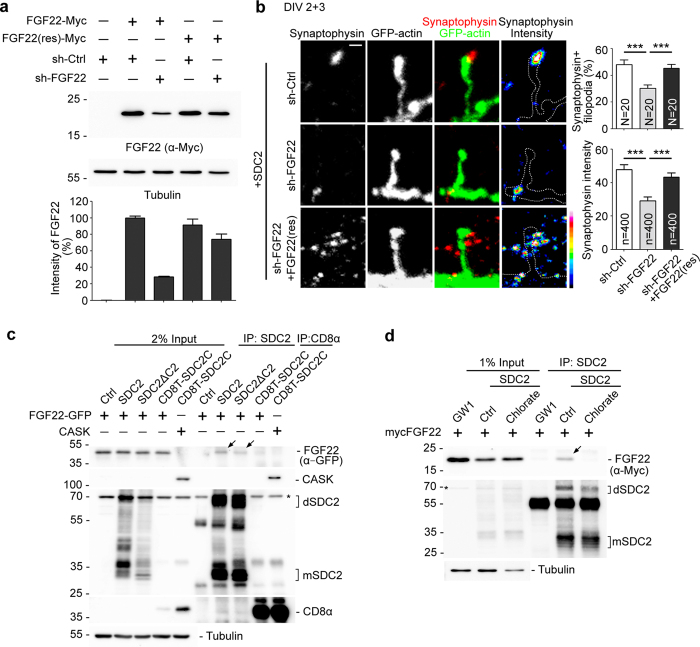
FGF22 interacts with SDC2 via heparan sulfate conjugates and contributes to SDC2-induced presynaptic differentiation. (**a**) The knockdown effect of sh-FGF22. Neuro2A cells were transfected with various plasmids as indicated and subjected to immunoblotting using indicated antibodies. FGF22(res) is a silent mutant of FGF22 resistant to sh-FGF22 knockdown. Lower panel indicates relative intensity of Myc-tagged FGF22 normalized with tubulin. Data represent mean plus SEM. (**b**) Knockdown of FGF22 impairs the presynaptic differentiation induced by SDC2 at 5 DIV. The heat maps show the intensities of synaptophysin. Four hundred filopodia collected from 20 neurons were analyzed for each group. Samples were collected from two independent experiments. Error bar indicates mean plus SEM. ***P < 0.001. Scale bar: 1 μm. (**c**) The extracellular domain of SDC2 is required for FGF22 interaction. Neuro2A cells were co-transfected with indicated constructs and subjected to immunoprecipitation and immunoblotting using the indicated antibodies. (**d**) Inhibition of heparan sulfation by sodium chlorate (30 mM) impairs SDC2-FGF22 interaction. dSDC2: SDC2 dimer; mSDC2: SDC2 monomer. Arrows indicate the coprecipitated FGF22. Asterisk indicates the non-specific signal.

We then knocked down endogenous FGF22 to investigate whether FGF22 is indeed involved in SDC2-induced synaptophysin accumulation. An artificial shRNA, sh-FGF22, was used to downregulate FGF22 expression (Fig. 4a). A FGF22 silent mutant, FGF22(res), that is resistant to sh-FGF22 (Fig. 4a) was also generated. Compared with a non-silencing control sh-Ctrl, expression of sh-FGF22 noticeably reduced the percentage of SDC2-induced synaptophysin-positive filopodia, as well as the intensity of individual synaptophysin puncta associated with SDC2-induced dendritic filopodia (Fig. 4b). These effects were specific to FGF22 knockdown because synaptophysin accumulation induced by postsynaptic SDC2 was rescued by coexpression of FGF22(res) mutant (Fig. 4b). These results suggest that FGF22 is critical for SDC2 to promote accumulation of presynaptic synaptophysin.

### Extracellular domain and heparan sulfate modification of SDC2 are required for the interaction with FGF22

We then evaluated our model by mapping the motifs of SDC2 required to induce transsynaptic signaling. SDC2 binding partners and their related functions are summarized in Fig. 1a (see Fig. 1b for the SDC2 constructs used in this report). If SDC2 indeed binds and targets FGF22 to dendritic filopodia and spines, we expected that the ectodomain of SDC2 would be required for FGF22 binding and targeting because SDC2 has a heparan sulfate modification at its ectodomain. To examine this possibility, we first investigated involvement of the SDC2 ectodomain in the interaction with FGF22. CD8T-SDC2C–a fusion of the ectodomain and transmembrane domain of CD8 and the cytoplasmic domain of SDC2 (Fig. 1b)–was used for coimmunoprecipitation. Because both SDC2 and CD8 form dimer through the transmembrane domain, CD8T-SDC2C is expected to form dimer, which is similar to SDC2. We found that FGF22 coprecipitated with WT SDC2, as well as SDC2ΔC2 (a SDC2 mutant lacking the C2 motif), but not CD8T-SDC2C (Fig. 4c). We noticed that SDC2ΔC2 seemed to precipitate less FGF22 than WT SDC2, but because SDC2ΔC2 presented multiple bands on the blot (as did WT SDC2), it was impossible for us to effectively quantify coimmunoprecipitation levels. Lack of interaction between CD8T-SDC2C and FGF22 is less likely due to misfolding of chimera proteins because CD8T-SDC2C was able to precipitate CASK, a C2-binding protein (Fig. 4c).

We further investigated the involvement of heparan sulfate modification in the interaction between SDC2 and FGF22 by addition of chlorate into cultures. Sulfation is one of the essential reactions of heparan sulfate biosynthesis. Chlorate inhibits the formation of 3′ phosphoadenosine-5′-phosphosulfate—the high energy sulfate donor for cellular sulfate reactions—and consequently prevents sulfation in heparan sulfate biosynthesis^30^,^31^. Through inhibition of heparan sulfate biosynthesis, chlorate treatment disrupts the activity of heparan sulfate proteoglycans to transduce FGF2 signaling^32^. Based on this rationale, we examined whether chlorate treatment disrupts coimmunoprecipitation of SDC2 and FGF22. Indeed, addition of chlorate completely abolished the coimmunoprecipitation of FGF22 with SDC2 (Fig. 4d). We also performed the coimmunoprecipitation using HEK293T cells, but failed to find an association between SDC2 and FGF22. It seems very likely that neuron-specific glycosylation influences the association of SDC2 and FGF22.

### Both the extracellular and C2 domains of SDC2 are involved in presynaptic maturation

**Figure 5:**
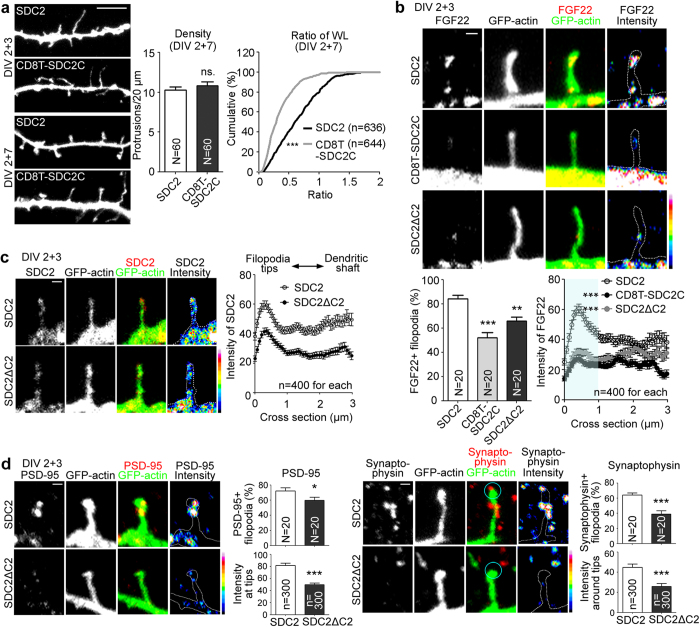
Both the ectodomain and cytoplasmic tail of SDC2 contribute to SDC2-induced synaptic differentiation. (**a**) CD8T-SDC2C does not induce the F-S transition. The protrusion density and the ratio of width to length of individual protrusions are shown. (**b**) CD8T-SDC2C and the SDC2ΔC2 mutant reduce FGF22-mCherry signals at filopodial tips. The percentage of FGF22-positive filopodia and the line scanning for the FGF22 intensity along filopodia are shown. The light blue area in the chart was used for statistical analysis. (**c**) Expression patterns of SDC2 and the SDC2ΔC2 mutant. The SDC2ΔC2 mutant displayed lower filopodial targeting. Line scanning from the tips of filopodia to dendritic shafts and the average intensity of SDC2 immunoreactivity at filopodial tips are shown. (**d**) Expression of the SDC2ΔC2 mutant decreased PSD-95 accumulation and surrounding synaptophysin at filopodial tips. Heat maps show the intensities of indicated proteins. Samples were collected from two independent experiments. In (**a**), N, the number of analyzed dendrites, n, the number of analyzed dendritic protrusions; in (**b–d**), N, the number of analyzed neurons; n, the number of analyzed dendritic protrusions. Data represent the mean plus SEM. *P < 0.05; **P < 0.01; ***P < 0.001; ns, not significant. Scale bar: (**a**) 5 μm; (**b–d**) 1 μm.

The contributions of different SDC2 domains in presynaptic differentiation were further investigated. We first examined the ability of CD8T-SDC2C to promote dendritic spine formation. Because CD8T-SDC2C contains the C1 motif–a region involved in filopodia formation (Fig. 1a)–CD8T-SDC2C was able to promote dendritic filopodia formation at 5 DIV (Fig. 5a); though CD8T-SDC2C-induced filopodia did not transform into dendritic spines at 9 DIV (Fig. 5a). Consistent with the failure to bind FGF22, CD8T-SDC2C could not promote filopodial targeting of FGF22, as the percentage of FGF22-positive filopodia and the FGF22 intensity at the filopodial tips were both reduced (Fig. 5b). These data suggest that interaction of the ectodomain of SDC2 with FGF22 is required for filopodial targeting of FGF22. These results also imply the involvement of the SDC2 ectodomain in the F-S transition.

Although SDC2ΔC2 interacted with FGF22 (Fig. 4c), we found that SDC2ΔC2 could not target FGF22 to filopodial tips (Fig. 5b), suggesting that the C2 motif, i.e. the CASK binding site, still indirectly influences FGF22 targeting. To further investigate the role of the C2 motif in presynaptic maturation, we performed two additional experiments. Our previous studies had indicated that the SDC2ΔC2 mutant promotes filopodia formation at 5 DIV^18^,^22^. Consistent with that ability to promote filopodia formation, here we found that both SDC2 and SDC2ΔC2 show a filopodial targeting pattern (Fig. 5c), although SDC2ΔC2 exhibited a lower intensity at filopodia compared to WT SDC2 (Fig. 5c). Moreover, the ability of SDC2ΔC2 to induce accumulation of postsynaptic PSD-95 and presynaptic synaptophysin was much weaker than that of SDC2 (Fig. 5d). The results suggest that although the C2 motif of SDC2 is not involved in FGF22 binding, it is still critical for both post- and pre-synaptic maturation.

### CaMKII and KIF17 regulate FGF22 targeting to SDC2-induced filopodial tips

**Figure 6:**
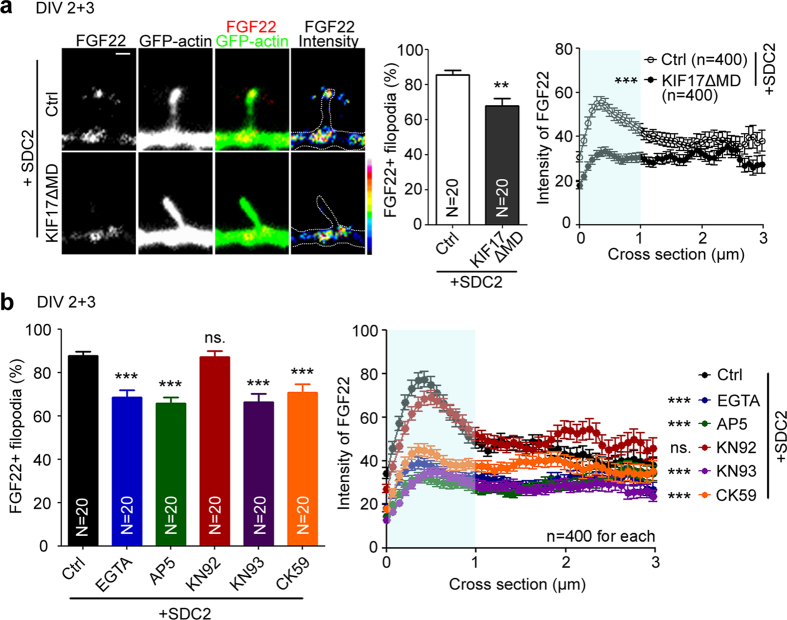
KIF17 and CaMKII are required for SDC2-mediated filopodial targeting of FGF22. (**a**) The KIF17ΔMD mutant reduces FGF22-mCherry distribution to filopodial tips. (**b**) Impairment of calcium influx and CaMKII inactivation reduces the filopodial distribution of FGF22-mCherry. Cultured rat hippocampal neurons were transfected with indicated plasmids at 2 DIV and subjected to immunostaining at 5 DIV. 1 mM EGTA, 100 μM AP5, 5 μM KN92, 5 μM KN93 and 10 μM CK59 were added into cultured neurons for 2 days before harvesting. The results of the percentages of FGF22-positive filopodia and line scanning from the tips of filopodia to dendritic shafts are shown. The light blue area in the chart was used for statistical analysis. Samples were collected from two independent experiments. Four hundred filopodia collected from 20 neurons were analyzed for each group. Error bar indicates mean plus SEM. **P < 0.01; ***P < 0.001; ns, not significant. Scale bar: 1 μm.

We then investigated how the C2 motif regulates FGF22 targeting. The C2 region of SDC2 interacts with the PDZ domain of CASK^12^. CASK is able to use its N-terminal CaMK-like and L27 domains to interact with MINT1 and mLIN7, respectively^33^,^34^, which further link the SDC2-CASK complex to the motor protein KIF17^27^ and NMDAR^22^. These interactions and functions are summarized in Fig. 1a. We previously showed that SDC2 expression facilitates dendritic filopodial targeting of NMDAR^22^. In addition, KIF17 also participates in synaptic targeting of FGF22^26^, though the mechanism is unclear. Based on our observations and information in the literature, we speculated that SDC2 links FGF22 to the KIF17 motor via the CASK-mLIN7-MINT1 tripartite complex, thereby regulating FGF22 targeting to filopodail tips. C2 deletion disrupts the association of the SDC2-FGF22 complex with the CASK-mLIN7-MINT1 tripartite complex and KIF17, hence attenuating filopodial targeting of FGF22. To investigate this scenario, we assessed whether KIF17 is involved in SDC2-dependent FGF22 targeting. We inactivated KIF17 by expressing a motor domain-deleted mutant KIF17ΔMD^26^,^35^. In SDC2-transfected neurons, KIF17ΔMD noticeably reduced the percentage of FGF22-positive filopodia and the intensity of FGF22 puncta in filopodial tips (Fig. 6a). Our results support the notion that KIF17 regulates SDC2-mediated FGF22 targeting.

A previous study showed that CaMKII receives synaptic calcium influx signals and phosphorylates KIF17. It then reduces the interaction of KIF17 and MINT1^36^. Disruption of KIF17-MINT1 interaction by CaMKII phosphorylation results in the release of the cargo from its microtubule-based transport machinery to local dendritic spines^36^. We have also shown that the SDC2-CASK-mLIN7 complex facilitates filopodial targeting of NMDAR, thus increasing the sensitivity of dendritic filopodia to neurotransmission and leading to calcium influx at postsynaptic sites^22^. We were interested to determine whether CaMKII and calcium signaling through NMDAR further promote the targeting of FGF22 to filopodial tips. Two CaMKII inhibitors, KN93 and CK59, were applied to SDC2-transfected neurons. KN92, an inactive analog of KN93, was included as control. Compared with KN92, we found that both KN93 and CK59 reduced the percentages of FGF22-positive filopodia and the intensities of FGF22 puncta in filopodia (Fig. 6b). Therefore, inactivation of CaMKII does reduce SDC2-mediated FGF22 targeting to dendritic filopodia.

To test the roles of calcium influx and NMDAR, we applied EGTA and AP5 (a NMDAR antagonist) to SDC2-transfected cultures. Compared with a vehicle control, both EGTA and AP5 reduced the percentages of FGF22-positive filopodia and the intensities of FGF22 puncta at filopodial tips (Fig. 6b). The effects of EGTA and AP5 were comparable to those of KN93 and CK59 (Fig. 6b). Taken together, our data suggest that calcium influx via NMDAR may activate CaMKII to further promote KIF17-mediated filopodial targeting of FGF22.

### FGF22 is critical for the SDC2-induced F-S transition

**Figure 7:**
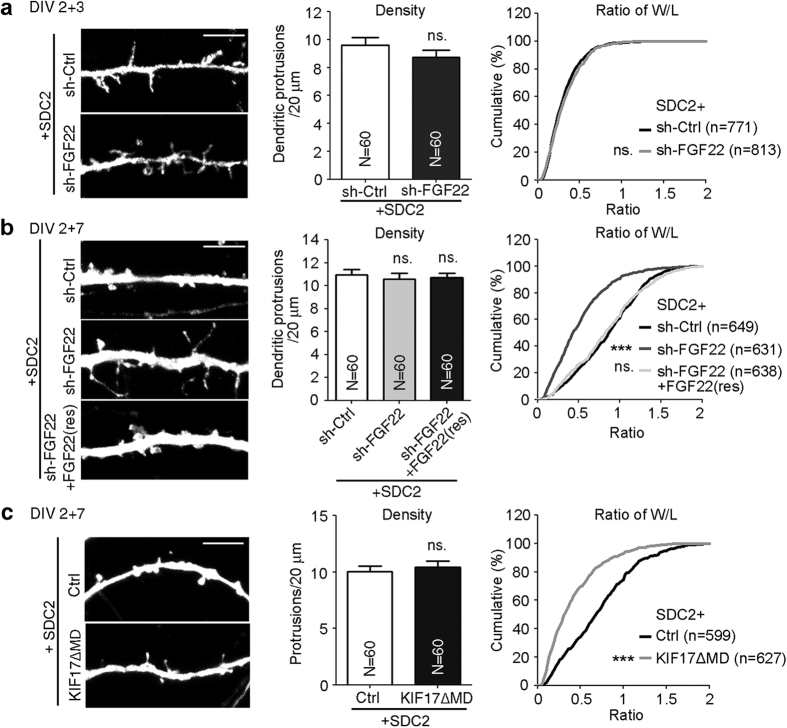
FGF22 triggers the F-S transition induced by SDC2. Cultured rat hippocampal neurons were co-transfected with various plasmids at 2 DIV and subjected to immunostaining at 5 or 9 DIV as indicated. (**a**) FGF22 knockdown does not influence filopodia formation. (**b**) FGF22 knockdown impairs the F-S transition. (**c**) The KIF17ΔMD mutant impairs the F-S transition of SDC2-induced filopodia. Representative images, quantification of protrusion density and the W/L ratio of dendritic protrusions are shown. The sample sizes of analyzed dendrites (N) and dendritic protrusions (n) are indicated. Samples were collected from two independent experiments. Data represent the mean plus SEM. ***P < 0.001; ns, not significant. Scale bar: 5 μm.

The aforementioned data suggest that at the dendritic filopodia-forming stage, FGF22 can be targeted to filopodial tips and promotes accumulation of the presynaptic marker synaptophysin. Meanwhile, NMDAR also targets to dendritic filopodial tips^22^. We hypothesized that presynaptic maturation induced by FGF22 provides a positive feedback signal to facilitate the F-S transition through NMDAR activation. To investigate this possibility, cultured hippocampal neurons were co-expressed with SDC2 and sh-FGF22 at 2 DIV and immunostaining was performed at 5 (Fig. 7a) and 9 DIV (Fig. 7b) to monitor filopodia and spine formation, respectively. Compared with the control shRNA, the density and ratio of width to length (W/L) of SDC2-induced filopodia were not altered by FGF22 knockdown at 5 DIV (Fig. 7a), suggesting that FGF22 knockdown has no obvious effect on dendritic filopodia formation. At 9 DIV, FGF22 knockdown still did not alter the density of dendritic protrusions (Fig. 7b). However, FGF22-knockdown neurons exhibited a narrower width of the filopodial protrusion head and a longer protrusion length, which reflected a lower W/L ratio (Fig. 7b, right panel). The effect of sh-FGF22 on the W/L ratio was specifically due to FGF22 knockdown because co-expression of the FGF22(res) silent mutant restored the W/L ratio to values similar to the sh-Ctrl control (Fig. 7b, right panel). These results indicate that reduction of FGF22 impairs the F-S transition.

We further examined whether the F-S transition is altered by KIF17ΔMD, which reduced filopodial targeting of FGF22 (Fig. 6a). Similar to the results of FGF22 knockdown, protrusion density was not affected by the KIF17ΔMD mutant (Fig. 7c), but the W/L ratio was noticeably lower in KIF17ΔMD mutant-expressing neurons (Fig. 7c, right panel). These data suggest the involvement of KIF17 in the F-S transition and are also consistent with the role of FGF22 in the F-S transition.

## Discussion

**Figure 8:**
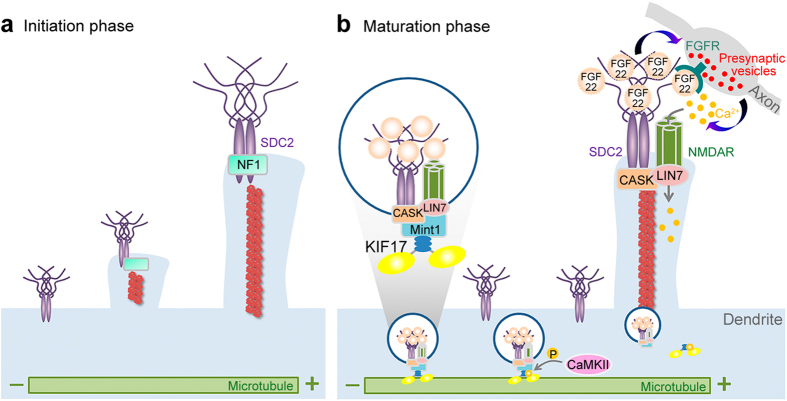
Bidirectional transsynaptic signaling promotes excitatory synapse formation. During development, neurons undergo filopodia formation ((**a)** initiation) and pre- and post-synaptic differentiation ((**b**) maturation) to form excitatory synapses. At the initiation stage, expression of SDC2 induces robust filopodia formation by interacting with neurofibromin encoded by the neurofibromatosis type I (*Nf1*) gene. Our previous study showed that SDC2 interacts with neurofibromin to increase cAMP concentration and induce ENA/VASP phosphorylation. Consequently, SDC2 promotes F-actin bundling and filopodia formation. For synapse maturation, postsynaptic FGF22 and presynaptic neurotransmission coordinate to form a positive feedback loop for pre- and post-synaptic differentiation. FGF22 binds to the ectodomain of SDC2 and is co-transported with NMDAR via the CASK-mLIN7-MINT1 adaptor and KIF17 motor. At the tips of filopodia, FGF22 is presented to presynaptic FGFR and promotes presynaptic vesicle accumulation. It consequently increases the probability of neurotransmitter release and activates NMDAR at the postsynaptic site. Calcium influx through NMDAR further activates CaMKII to phosphorylate KIF17 and induce SDC2-CASK-mLIN7-NMDAR release from the microtubule to the area with a higher concentration of calcium. These sequential regulations establish a positive feedback mechanism to coordinate and accelerate pre- and post-synaptic differentiation. Using this positive feedback, dendritic filopodia can then transform into mature dendritic spines.

In this study, we show that FGF22 is an effector of SDC2 to induce presynaptic differentiation. The ectodomain of SDC2 interacts with FGF22 and facilitates FGF22 targeting to dendritic filopodial tips. FGF22 is then presented to presynaptic sites to promote presynaptic differentiation, as indicated by the accumulation of synaptophysin, a presynaptic vesicle protein. With the aggregation of presynaptic vesicles, the frequency and probability of stimulating the corresponding postsynaptic sites are then increased. Because SDC2 also brings NMDAR to filopodial tips through the interaction with the CASK-mLIN7-MINT1 complex^22^, filopodial distribution of NMDAR guarantees the susceptibility of SDC2-induced filopodia to neurotransmitter (glutamate) stimulation. Calcium influx via NMDAR then triggers F-actin cytoskeleton rearrangement and promotes the F-S transition^22^. Thus, by combining the retrograde signal of FGF22 and anterograde neurotransmission, the positive transsynaptic feedback loop established by the SDC2-associated complex coordinates the differentiation of both pre- and post-synaptic termini (Fig. 8), explaining why postsynaptic expression of SDC2 triggers both post- and pre-synaptic differentiation.

It is interesting that overexpression of SDC2 is sufficient to promote dendritic spine formation in immature neurons. Based on previous studies and this report, we suggest that the ability of SDC2 to associate with several different proteins–including FGF22, CASK, mLIN7, MINT1, KIF17 and NMDAR–makes SDC2 one of key molecules for promoting dendritic spinogenesis (Fig. 8). In young cultures, i.e. before 9 DIV, levels of endogenous SDC2 are very low; in fact, under the detection threshold of RT-PCR^13^. FGF22 and NMDAR are already present in these young cultures but, in the absence of SDC2, they do not interact to coordinate pre- and post-synaptic differentiation. Thus, for the intrinsic process, dendritic filopodia formed in immature neurons are transient and unstable. However, in the presence of SDC2, FGF22 and NMDAR functionally interact to stabilize pre- and post-synaptic interactions and further promote the F-S transition.

Note that in SDC2-induced dendritic spinogenesis, filopodia and spine formation can be clearly separated, allowing us to easily dissect the molecular regulation of these two sequential stages. SDC2-induced filopodia formation is not neuron-specific; it can also happen in non-neuronal cells, such as HEK cells^18^. The interaction between the SDC2 C1 motif and neurofibromin delivers the signal to activate PKA, to phosphorylate ENA/VASP and consequently to promote F-actin bundling and filopodia formation^18^. In contrast, SDC2-induced dendritic spine formation is neuron-specific^37^. Based on our previous and current studies^22^,^37^, we suggest that NMDAR-mediated neuronal activation is critical for achieving this neuron-specific event. NMDAR located at the filopodia receives the feedback signal from the presynaptic site to induce calcium influx. Our previous study suggested that NMDAR-mediated calcium influx regulates F-actin rearrangement through cytoskeleton regulators, such as gelsolin^22^. Activation of gelsolin promotes F-actin severing and thus shortens the length of dendritic protrusions, which is one of the morphological features of the F-S transition. In this report, we further suggest that CaMKII downstream of NMDAR plays a role in regulation of KIF17-dependent dendritic filopodial targeting of FGF22. Because CaMKII and KIF17 are downstream of NMDAR in release of FGF22, they are expected to be more important for enhancing FGF22 targeting to dendritic filopodia that have already received presynaptic stimulation. Consistent with this speculation, FGF22 is not involved in formation of SDC2-induced dendritic filopodia (Fig. 7a), although FGF22 is required for dendritic spine maturation (Fig. 7b). Similarly, KIF17 is also required for dendritic spine maturation (Fig. 7c). Although our data suggest that the effect of FGF22 on postsynaptic maturation is likely mediated by the feedback of presynaptic neurotransmission, we cannot rule out the possibility that postsynaptic FGF22 also cell-autonomously regulates postsynaptic maturation. FGF22 has been shown to release from postsynaptic site to promote presynaptic maturation. However, it is still possible that presynaptically-released FGF22 binds to postsynaptic SDC2 and then is presented back to presynaptic FGFR for presynaptic differentiation. More investigations need to be performed to address this possibility.

In this report, we unexpectedly found that SDC2 interacts with FGF22 in Neuro2A cells but not HEK293 cells. Because chlorate treatment abolished the interaction between SDC2 and FGF22 in Neuro2A cells, it suggests that cell-type specific glycosylation regulates the SDC2-FGF22 interaction. Glycosylation is a complex process that varies from cells to cells^38^,^39^. For heparan sulfate, a combination of epimerization, deacetylation and multiple sulfation reactions generates a great deal of diversity (up to 10^36^ types) of heparan sulfate isoforms^38^,^40^. Moreover, tissue-specific expression and ligand binding specificity have also been reported for heparan sulfate^40^,^41^,^42^. Thus, it is reasonable to speculate that neuron-specific glycosylation influences the interaction between SDC2 and FGF22. Although currently a difficult proposition, it would be very intriguing to explore in the future how neuron-specific glycosylation controls the SDC2-FGF22 interaction and synaptic differentiation.

In conclusion, our study suggests that the postsynaptic SDC2-associated complex establishes a complex yet elegant feedback regulatory mechanism to coordinate pre- and post-synaptic differentiation, which contains both retrograde and anterograde signals.

## Methods

### Antibodies and chemicals

Antibodies and chemicals used in this report are as follows: rabbit polyclonal syndecan-2 antibody (syndecan-2G, 1:1000)^18^; mouse monoclonal PSD-95 (K28/43, MABN68, 1:1000, Millipore)^43^; mouse monoclonal synaptophysin (S5768, 1:1000, Sigma)^44^; mouse monoclonal Tubulin (B-5-1-2, 1:5000, Sigma)^43^; chicken polyclonal GFP (ab13970, 1:5000, Abcam)^43^; mouse monoclonal GFP (JL-8, 632381, 1:2000, Clontech); rabbit polyclonal Cherry (632496, 1:1000, Clontech); rat monoclonal CD8α (14-0081, 1:1000, eBioscience)^45^; mouse monoclonal VCP (612183, 1:1000, BD Biosciences)^46^; mouse monoclonal Myc (9B11, #2276, 1:1000, Cell Signaling Technology)^43^; mouse monoclonal CASK (K56A, MAB5230, 1:500, Millipore)^22^; mouse monoclonal Oyster-550-conjugated synaptotagmin (105 311C3, 1:400, Synaptic System); Alexa Fluor-488- and-555-conjugated secondary antibodies (Invitrogen); Sodium chlorate (Sigma); Ethylene glycol tetraacetic acid (EGTA, Sigma); 2-amino-5-phosphonopentanoic acid (AP5; Sigma); KN-92 (Cayman chemical); KN-93 (Sigma); CK-59 (Calbiochem).

### Plasmid construction

SDC2, SDC2ΔC2, SDC2 RNAi knockdown, FGF22-mCherry, FGF22-GFP and FGF22 RNAi knockdown constructs have been previously described (Umemori *et al.*^24^; Lin *et al.*^18^; Terauchi *et al.*^25^; Terauchi *et al.*^26^). For myc-tagged FGF22, full-length FGF22 cDNA was subcloned into the GW1-myc vector. To generate the silent mutant resistant to sh-FGF22, site-directed mutagenesis was performed using the following oligonucleotide: 5′-GGCAAGG*G*AGACGGAC*T*CGACGGCA*T*CAA-3′ (bases in italics indicate the mutated sites). For CD8T-SDC2C fusion, the cDNA fragment corresponding to the first 220 amino acid residues of mouse CD8α was PCR amplified and fused with the fragment containing the last 32 amino acid residues of SDC2. An extra BglII site was inserted between the fragments of CD8α and SDC2 for cloning purposes. GFP-actin was purchased from Clontech.

### Hippocampal neuronal culture, transfection and neuronal treatment

Hippocampal neurons from embryonic day 18–19 SD rat embryos were cultured and transfected as previously described (Lin *et al.*^18^; Chao *et al.*^23^; Hu and Hsueh^22^). To examine intrinsic synapse formation, a density of 200,000 neurons per well was transfected at 12 DIV and subjected to immunostaining at 18 DIV. To study the effect of SDC2 on synapse maturation, neurons at a density of 300,000 cells per well were transfected with SDC2 at 2 DIV, and immunostaining was performed at 5 DIV for filopodial morphology and at 9 DIV for spine morphology. The GFP-actin construct was cotransfected with indicated plasmids into neurons to outline the cellular morphology. To study the effect of calcium influx on FGF22 targeting, EGTA (1 mM) and AP5 (100 μM) were added into neurons for 48 hrs before harvesting. To examine the effect of CaMKII activity on FGF22 targeting, CaMKII inhibitor KN93 (5 μM) and CK59 (10 μM) were also added into neurons for 48 hrs before harvesting. KN92 (5 μM) was the negative control of KN93.

### Acquisition of immunofluorescence images

Cells were washed with phosphate-buffered saline (PBS) and fixed with 4% paraformaldehyde and 4% sucrose in PBS for 10 min, followed by permeabilization with 0.2% Triton X-100 in PBS or cold methanol for 15 min (for synaptophysin and synaptotagmin) at room temperature. After blocking with 10% bovine serum albumin (BSA), cells were incubated with primary antibodies diluted in PBS containing 3% BSA and 0.1% horse serum at 4 °C overnight, followed by extensive washes with PBS, and incubated with secondary antibodies conjugated with Alexa Fluor-488 and-555 (Invitrogen) for 2 hrs at room temperature. The images were acquired at room temperature using a confocal microscope (LSM700; Carl Zeiss) equipped with a 63×/NA 1.4 oil objective lens and Zen 2009 (Carl Zeiss) acquisition and analysis software. For quantitation, the same set of experimental samples was acquired under the same confocal microscopy settings. Post-acquisition adjustment was avoided. To minimize personal bias, a blind test was performed for image acquisition.

### Synaptotagmin antibody uptake assay

To monitor the functional recycling vesicles in presynaptic termini, synaptotagmin antibody uptake assays were performed in living neurons^28^,^29^. Oyster-550-conjugated synaptotagmin antibody was added into cultures for 1 h at 37 °C. Cells were then washed to remove free synaptotagmin antibody and subjected to immunofluorescence staining as described above.

### Analyses of dendritic protrusions and synaptic protein distribution

To characterize the dendritic protrusions, three 20 μm-long segments of dendrites starting 20 μm away from the soma of each neuron were selected to analyze the following four parameters: density, width, length and width/length (W/L) ratio of the protrusions. The analyses were manually performed using ImageJ software (NIH). The filopodial tip was defined by the region within 1 μm of the ends of filopodia. To quantify the distribution and immunoreactivities of postsynaptic PSD-95 and FGF22 along dendritic protrusions, line scanning using ImageJ was performed. A line 0.5 μm wide starting from the top of the dendritic protrusion and ending at the dendritic shaft was drawn to quantify protein intensities. Total immunoreactivities within 1 μm of the top of protrusions defined as the postsynaptic region were summed for statistical analysis. To quantify the presynaptic protein synaptophysin and synaptotagmin, a circle of 1 μm diameter was drawn at the top of the dendritic protrusion (Fig. 2a, right panel), and immunoreactivities were quantified using ImageJ. Protrusions with an intensity >20 units were categorized as protein-positive.

### Transfection, immunoblotting and immunoprecipitation of N2A cells

Mouse neurobalstoma Neuro-2A (N2A) cells (ATCC CCL-131) were transfected with the indicated plasmids using Lipofectamine 2000 (Invitrogen) according to the manufacturer’s instructions. To inhibit sulfation, cells were treated with 30 mM sodium chlorate for 24 hrs before harvesting. For immunoblotting analysis, the cells were washed with PBS and lysed directly with SDS sample buffer. For immunoprecipitation analysis, the cells were washed with PBS and solubilized in lysis buffer (PBS, pH 7.4, 1% Triton X-100, 2 mM PMSF, 2 μg/ml aprotinin, 2 μg/ml leupeptin, 2 μg/ml pepstatin and 10 μM MG132) at 4 °C for 30 min. The lysates were centrifuged at 15,000 × g for 30 min to remove the cell debris. The soluble extract was subjected to immunoprecipitation using specific antibodies, followed by immunoblotting.

### Animals and housing

All animal experiments were performed with the approval of and in strict accordance with the guidelines of the Academia Sinica Institutional Animal Care and Utilization Committee and the Republic of China Council of Agriculture Guidebook for the Care and Use of Laboratory Animals. Pregnant rats were housed individually and sacrificed using CO_2_ inhalation. All efforts were made to minimize animal suffering and to reduce the number of animals required.

### Quantification and statistical analysis

The data were collected and analyzed blind by having other members of the laboratory relabel the samples. For each experiment, 20 neurons were randomly collected from two independent experiments for quantification. To analyze dendritic spine density, three clearly recognizable dendrites of each neuron were used for quantification. To monitor protein distribution in or surround dendritic protrusions, 400 dendritic protrusions were analyzed for each group. The results of the protrusion density and distribution of synaptic proteins were analyzed with an unpaired Student’s t test using GraphPad Prism 5.0 (GraphPad Software). For more than two group comparisons, one-way ANOVA with Tukey’s multiple comparison post-hoc test was performed. All of the results shown in the cumulative probability distribution were analyzed using the Kolmogorov-Smirnov test Kirkman, T.W. (1996). Statistics to use http://www.physics.csbsju.edu/stats/. A P-value of less than 0.05 was considered significant.

## Additional Information

**How to cite this article**: Hu, H.-T. *et al.* Postsynaptic SDC2 induces transsynaptic signaling via FGF22 for bidirectional synaptic formation. *Sci. Rep.* **6**, 33592; doi: 10.1038/srep33592 (2016).

## References

[b1] Graf E. R., Zhang X., Jin S. X., Linhoff M. W. & Craig A. M. Neurexins induce differentiation of GABA and glutamate postsynaptic specializations via neuroligins. Cell 119, 1013–1026 (2004). 15620359 10.1016/j.cell.2004.11.035 PMC2826211

[b2] Chih B., Engelman H. & Scheiffele P. Control of excitatory and inhibitory synapse formation by neuroligins. Science 307, 1324–1328 (2005). 15681343 10.1126/science.1107470

[b3] Chubykin A. A. *et al.* Activity-dependent validation of excitatory versus inhibitory synapses by neuroligin-1 versus neuroligin-2. Neuron 54, 919–931 (2007). 17582332 10.1016/j.neuron.2007.05.029 PMC3738748

[b4] Liu A. *et al.* Neuroligin 1 regulates spines and synaptic plasticity via LIMK1/cofilin-mediated actin reorganization. J Cell Biol 212, 449–463 (2016). 26880202 10.1083/jcb.201509023 PMC4754719

[b5] Wittenmayer N. *et al.* Postsynaptic Neuroligin1 regulates presynaptic maturation. Proc Natl Acad Sci USA 106, 13564–13569 (2009). 19628693 10.1073/pnas.0905819106 PMC2726414

[b6] Stan A. *et al.* Essential cooperation of N-cadherin and neuroligin-1 in the transsynaptic control of vesicle accumulation. Proc Natl Acad Sci USA 107, 11116–11121 (2010). 20534458 10.1073/pnas.0914233107 PMC2890764

[b7] Aiga M., Levinson J. N. & Bamji S. X. N-cadherin and neuroligins cooperate to regulate synapse formation in hippocampal cultures. J Biol Chem 286, 851–858 (2011). 21056983 10.1074/jbc.M110.176305 PMC3013044

[b8] Lai K. O. & Ip N. Y. Synapse development and plasticity: roles of ephrin/Eph receptor signaling. Curr Opin Neurobiol 19, 275–283 (2009). 19497733 10.1016/j.conb.2009.04.009

[b9] Hruska M. & Dalva M. B. Ephrin regulation of synapse formation, function and plasticity. Mol Cell Neurosci 50, 35–44 (2012). 22449939 10.1016/j.mcn.2012.03.004 PMC3631567

[b10] Klein R. Bidirectional modulation of synaptic functions by Eph/ephrin signaling. Nat Neurosci 12, 15–20 (2009). 19029886 10.1038/nn.2231

[b11] Linhoff M. W. *et al.* An unbiased expression screen for synaptogenic proteins identifies the LRRTM protein family as synaptic organizers. Neuron 61, 734–749 (2009). 19285470 10.1016/j.neuron.2009.01.017 PMC2746109

[b12] Hsueh Y. P. *et al.* Direct interaction of CASK/LIN-2 and syndecan heparan sulfate proteoglycan and their overlapping distribution in neuronal synapses. J Cell Biol 142, 139–151 (1998). 9660869 10.1083/jcb.142.1.139 PMC2133027

[b13] Ethell I. M. & Yamaguchi Y. Cell surface heparan sulfate proteoglycan syndecan-2 induces the maturation of dendritic spines in rat hippocampal neurons. J Cell Biol 144, 575–586 (1999). 9971750 10.1083/jcb.144.3.575 PMC2132915

[b14] Bernfield M. *et al.* Functions of cell surface heparan sulfate proteoglycans. Annu Rev Biochem 68, 729–777 (1999). 10872465 10.1146/annurev.biochem.68.1.729

[b15] Filla M. S., Dam P. & Rapraeger A. C. The cell surface proteoglycan syndecan-1 mediates fibroblast growth factor-2 binding and activity. J Cell Physiol 174, 310–321 (1998). 9462693 10.1002/(SICI)1097-4652(199803)174:3<310::AID-JCP5>3.0.CO;2-R

[b16] Klass C. M., Couchman J. R. & Woods A. Control of extracellular matrix assembly by syndecan-2 proteoglycan. J Cell Sci 113 (Pt 3), 493–506 (2000). 10639336 10.1242/jcs.113.3.493

[b17] Hsueh Y. P. & Sheng M. Regulated expression and subcellular localization of syndecan heparan sulfate proteoglycans and the syndecan-binding protein CASK/LIN-2 during rat brain development. J Neurosci 19, 7415–7425 (1999). 10460248 10.1523/JNEUROSCI.19-17-07415.1999 PMC6782500

[b18] Lin Y. L., Lei Y. T., Hong C. J. & Hsueh Y. P. Syndecan-2 induces filopodia and dendritic spine formation via the neurofibromin-PKA-Ena/VASP pathway. J Cell Biol 177, 829–841 (2007). 17548511 10.1083/jcb.200608121 PMC2064283

[b19] Hsueh Y. P., Roberts A. M., Volta M., Sheng M. & Roberts R. G. Bipartite interaction between neurofibromatosis type I protein (neurofibromin) and syndecan transmembrane heparan sulfate proteoglycans. J Neurosci 21, 3764–3770 (2001). 11356864 10.1523/JNEUROSCI.21-11-03764.2001 PMC6762697

[b20] Grootjans J. J. *et al.* Syntenin, a PDZ protein that binds syndecan cytoplasmic domains. Proc Natl Acad Sci USA 94, 13683–13688 (1997). 9391086 10.1073/pnas.94.25.13683 PMC28366

[b21] Ethell I. M., Hagihara K., Miura Y., Irie F. & Yamaguchi Y. Synbindin, A novel syndecan-2-binding protein in neuronal dendritic spines. J Cell Biol 151, 53–68 (2000). 11018053 10.1083/jcb.151.1.53 PMC2189810

[b22] Hu H. T. & Hsueh Y. P. Calcium influx and postsynaptic proteins coordinate the dendritic filopodium-spine transition. Developmental neurobiology 74, 1011–1029 (2014). 24753440 10.1002/dneu.22181

[b23] Chao H. W., Hong C. J., Huang T. N., Lin Y. L. & Hsueh Y. P. SUMOylation of the MAGUK protein CASK regulates dendritic spinogenesis. J Cell Biol 182, 141–155 (2008). 18606847 10.1083/jcb.200712094 PMC2447900

[b24] Umemori H., Linhoff M. W., Ornitz D. M. & Sanes J. R. FGF22 and its close relatives are presynaptic organizing molecules in the mammalian brain. Cell 118, 257–270 (2004). 15260994 10.1016/j.cell.2004.06.025

[b25] Terauchi A. *et al.* Distinct FGFs promote differentiation of excitatory and inhibitory synapses. Nature 465, 783–787 (2010). 20505669 10.1038/nature09041 PMC4137042

[b26] Terauchi A. *et al.* Selective synaptic targeting of the excitatory and inhibitory presynaptic organizers FGF22 and FGF7. J Cell Sci 128, 281–292 (2015). 25431136 10.1242/jcs.158337 PMC4294774

[b27] Setou M., Nakagawa T., Seog D. H. & Hirokawa N. Kinesin superfamily motor protein KIF17 and mLin-10 in NMDA receptor-containing vesicle transport. Science 288, 1796–1802 (2000). 10846156 10.1126/science.288.5472.1796

[b28] Ammendrup-Johnsen I., Naito Y., Craig A. M. & Takahashi H. Neurotrophin-3 Enhances the Synaptic Organizing Function of TrkC-Protein Tyrosine Phosphatase sigma in Rat Hippocampal Neurons. J Neurosci 35, 12425–12431 (2015). 26354911 10.1523/JNEUROSCI.1330-15.2015 PMC4563035

[b29] Malgaroli A. *et al.* Presynaptic component of long-term potentiation visualized at individual hippocampal synapses. Science 268, 1624–1628 (1995). 7777862 10.1126/science.7777862

[b30] Humphries D. E. & Silbert J. E. Chlorate: a reversible inhibitor of proteoglycan sulfation. Biochem Biophys Res Commun 154, 365–371 (1988). 2969240 10.1016/0006-291x(88)90694-8

[b31] Safaiyan F. *et al.* Selective effects of sodium chlorate treatment on the sulfation of heparan sulfate. J Biol Chem 274, 36267–36273 (1999). 10593915 10.1074/jbc.274.51.36267

[b32] Quarto N. & Amalric F. Heparan sulfate proteoglycans as transducers of FGF-2 signalling. J Cell Sci 107 (Pt 11), 3201–3212 (1994). 7699017 10.1242/jcs.107.11.3201

[b33] Borg J. P. *et al.* Identification of an evolutionarily conserved heterotrimeric protein complex involved in protein targeting. J Biol Chem 273, 31633–31636 (1998). 9822620 10.1074/jbc.273.48.31633

[b34] Butz S., Okamoto M. & Sudhof T. C. A tripartite protein complex with the potential to couple synaptic vesicle exocytosis to cell adhesion in brain. Cell 94, 773–782 (1998). 9753324 10.1016/s0092-8674(00)81736-5

[b35] Guillaud L., Setou M. & Hirokawa N. KIF17 dynamics and regulation of NR2B trafficking in hippocampal neurons. J Neurosci 23, 131–140 (2003). 12514209 10.1523/JNEUROSCI.23-01-00131.2003 PMC6742138

[b36] Guillaud L., Wong R. & Hirokawa N. Disruption of KIF17-Mint1 interaction by CaMKII-dependent phosphorylation: a molecular model of kinesin-cargo release. Nat Cell Biol 10, 19–29 (2008). 18066053 10.1038/ncb1665

[b37] Hu H. T., Shih P. Y., Shih Y. T. & Hsueh Y. P. The Involvement of Neuron-Specific Factors in Dendritic Spinogenesis: Molecular Regulation and Association with Neurological Disorders. Neural Plast 2016, 5136286 (2016). 26819769 10.1155/2016/5136286 PMC4706964

[b38] Van Vactor D., Wall D. P. & Johnson K. G. Heparan sulfate proteoglycans and the emergence of neuronal connectivity. Curr Opin Neurobiol 16, 40–51 (2006). 16417999 10.1016/j.conb.2006.01.011

[b39] Rexach J. E., Clark P. M. & Hsieh-Wilson L. C. Chemical approaches to understanding O-GlcNAc glycosylation in the brain. Nature chemical biology 4, 97–106 (2008). 18202679 10.1038/nchembio.68 PMC3250351

[b40] Esko J. D. & Selleck S. B. Order out of chaos: assembly of ligand binding sites in heparan sulfate. Annu Rev Biochem 71, 435–471 (2002). 12045103 10.1146/annurev.biochem.71.110601.135458

[b41] Dietrich C. P., Nader H. B. & Straus A. H. Structural differences of heparan sulfates according to the tissue and species of origin. Biochem Biophys Res Commun 111, 865–871 (1983). 6220714 10.1016/0006-291x(83)91379-7

[b42] Maccarana M., Sakura Y., Tawada A., Yoshida K. & Lindahl U. Domain structure of heparan sulfates from bovine organs. J Biol Chem 271, 17804–17810 (1996). 8663266 10.1074/jbc.271.30.17804

[b43] Chen Y. K. & Hsueh Y. P. Cortactin-binding protein 2 modulates the mobility of cortactin and regulates dendritic spine formation and maintenance. J Neurosci 32, 1043–1055 (2012). 22262902 10.1523/JNEUROSCI.4405-11.2012 PMC6621164

[b44] Chivet M. *et al.* Exosomes secreted by cortical neurons upon glutamatergic synapse activation specifically interact with neurons. Journal of extracellular vesicles 3, 24722 (2014). 25398455 10.3402/jev.v3.24722 PMC4232649

[b45] Mochimaru H. *et al.* Suppression of alkali burn-induced corneal neovascularization by dendritic cell vaccination targeting VEGF receptor 2. Investigative ophthalmology &amp; visual science 49, 2172–2177 (2008). 18263815 10.1167/iovs.07-1396

[b46] Wang H. F. *et al.* Valosin-containing protein and neurofibromin interact to regulate dendritic spine density. J Clin Invest 121, 4820–4837 (2011). 22105171 10.1172/JCI45677 PMC3225986

